# Phylogenetic and antimicrobial resistance gene analysis of *Salmonella* Typhimurium strains isolated in Brazil by whole genome sequencing

**DOI:** 10.1371/journal.pone.0201882

**Published:** 2018-08-13

**Authors:** Fernanda Almeida, Amanda Aparecida Seribelli, Marta Inês Cazentini Medeiros, Dália dos Prazeres Rodrigues, Alessandro de MelloVarani, Yan Luo, Marc W. Allard, Juliana Pfrimer Falcão

**Affiliations:** 1 Departamento de Análises Clínicas, Toxicológicas e Bromatológicas–Faculdade de Ciências Farmacêuticas de Ribeirão Preto, Universidade de São Paulo, Av. do Café s/ nº, Ribeirão Preto, SP Brasil; 2 Centro de Laboratório Regional de Ribeirão Preto—Instituto Adolfo Lutz, Rua Minas, Ribeirão Preto, SP, Brasil; 3 Laboratório de Enterobactérias, FIOCRUZ/Fundação Instituto Oswaldo Cruz, Avenida Brasil, Pavilhão Rocha Lima, 3°andar, Manguinhos, Rio de Janeiro, RJ, Brasil; 4 Faculdade de Ciências Agrárias e Veterinárias, Universidade Estadual Paulista (UNESP), Jaboticabal, Brazil; 5 Division of Microbiology, Office of Regular Science, Center for Food Safety and Applied Nutrition, U.S. Food and Drug Administration, College Park, Maryland, United States of America; Cornell University, UNITED STATES

## Abstract

Whole genome sequencing (WGS) has been used as a powerful technology for molecular epidemiology, surveillance, identification of species and serotype, identification of the sources of outbreaks, among other purposes. In Brazil, there is relatively few epidemiological data on *Salmonella*. In this study, 90 *Salmonella* Typhimurium strains had their genome sequenced to uncover the diversity of *Salmonella* Typhimurium isolated from humans and food, between 1983 and 2013, from different geographic regions in Brazil based on single nucleotide polymorphism (SNP) analysis. A total of 39 resistance genes were identified, such as aminoglycoside, tetracycline, sulfonamide, trimethoprim, beta-lactam, fluoroquinolone, phenicol and macrolide, as well as the occurrence of point mutations in some of the genes such as *gyrA*, *gyrB*, *parC* and *parE*. A total of 65 (72.2%) out of 90 *S*. Typhimurium strains studied were phenotypically resistant to sulfonamides, 44 (48.9%) strains were streptomycin resistant, 27 (30%) strains were resistant to tetracycline, 21 (23.3%) strains were gentamicin resistant, and seven (7.8%) strains were resistant to ceftriaxone. In the *gyrA* gene, it was observed the following amino acid substitutions: Asp(87)→Gly, Asp(87)→Asn, Ser(83)→Phe, Ser(83)→Tyr. Phylogenetic results placed the 90 *S*. Typhimurium strains into two major clades suggesting the existence of a prevalent subtype, likely more adapted, among strains isolated from humans, with some diversity in subtypes in foods. The variety and prevalence of resistant genes found in these *Salmonella* Typhimurium strains reinforces their potential hazard for humans and the risk in foods in Brazil.

## Introduction

Foodborne diseases have a major impact on the economy and public health worldwide. Non-typhoidal *Salmonella* (NTS) is one of the most common causes of bacterial foodborne illnesses [1, 2]. It is estimated that NTS cause about 93.8 million annual cases of gastroenteritis and 155 thousand deaths per year worldwide [1].

Among the *Salmonella enterica* serovars, *Salmonella* Typhimurium (*S*. Typhimurium) is among the most frequent ones isolated worldwide [3]. From 2001 to 2007, this serovar was the most prevalent in the United States, Canada, Australia and New Zealand. In the same period, *S*. Typhimurium appeared as the second most prevalent serovar in Africa, Asia, Europe and Latin America, surpassed only by *S*. Enteritidis [3].

In Brazil, there are relatively little epidemiological data on *Salmonella* [4–7]. However, it is known that in the State of São Paulo, *S*. Typhimurium was the most commonly isolated serovar from human sources and the third most common from non-human sources before the 1990’s [4]. After this period, *S*. Typhimurium declined becoming the third most commonly isolated serovar from human and non-human sources in the period of 1991–1995 in São Paulo State in Brazil, with *S*. Enteritidis being the most isolated serovar in both sources and, *S*. I 4, (5), 12:i:- and *S*. Havana the second most isolated serovar in human and non-human sources, respectively [5]. Between 1996 and 2000, the isolation of *S*. Typhimurium declined even more from non-human sources [6]. However, between 1996 and 2003, this serovar was ranked as the second most commonly isolated serovar from human sources [7].

Epidemiological studies have been crucial to verify the relationship among pathogenic strains isolated from different sources, to elucidate contamination routes and to differentiate strains isolated from outbreaks and sporadic cases. Investigative capabilities have been greatly enhanced with the development and increasing feasibility of WGS as a molecular epidemiological tool [8–10]. Over the last few years there has been a substantial reduction in the costs of WGS making this technology economically viable as a routine tool for molecular epidemiology. WGS has also been used for detection of antibiotic resistance determinants [11, 12].

The use of antimicrobials is not recommended in cases of noninvasive *Salmonella* infections [13, 14]. However, in some cases, the antibiotic therapy might be necessary. The drug of choice for the treatment of *Salmonella* infections is typically ciprofloxacin due to its broad spectrum antimicrobial activity [14].

The extensive use of antimicrobials has led to increasing numbers of non-typhoidal *Salmonella* strains that are resistant to quinolones and exhibited reduced susceptibility to fluoroquinolones [15–17]. This reduced susceptibility can lead to treatment failures in some cases [18, 19]. Quinolone resistance is usually mediated by mutations in the quinolone resistance determining regions (QRDRs) of the *gyrA*, *gyrB*, *parC*, and *parE* genes that code for bacterial DNA gyrase leading to changes in the binding site of the antimicrobial to the enzyme [17, 20, 21]. Also, quinolone resistance may be due to the acquisition of plasmid-mediated quinolone resistance (PMQR) genes [22–24], such as the *qnr* genes that encodes a group of pentapeptide proteins that bind to DNA gyrase and prevent the action of quinolones, *qepA* gene, an quinolone efflux pump, *aac(6’)-Ib-cr* gene that encodes to the aminoglycoside acetiltranferase that can reduce susceptibility to ciprofloxacin and *oqxAB* genes, a multidrug resistance efflux pump [25].

In previous studies of our group, we typed *S*. Typhimurium strains isolated from humans and food between 1983 and 2013 in Brazil by Pulsed-field gel electrophoresis (PFGE), multiple-locus variable number of tandem repeats analysis (MLVA), enterobacterial repetitive intergenic consensus PCR (ERIC-PCR), CRISPR-multi-locus virulence sequence typing (CRISPR-MVLST) and Multilocus sequence typing (MLST). Moreover, the frequency of 12 virulence markers was assessed by PCR and the resistance profile against 12 antimicrobials was verified [26–28].

In this present work, WGS is used to uncover the diversity of *Salmonella* Typhimurium isolated from humans and food, between 1983 and 2013, from different geographic regions in Brazil. Additionally, WGS is used to verify the presence of antimicrobial resistance genes, as well as, the occurrence of mutations points in the *gyrA*, *gyrB*, *parC* and *parE* genes.

## Materials and methods

### Bacterial strains

A total of 90 *S*. Typhimurium strains were sequenced including: 42 strains from human clinical material such as diarrheic feces (n = 40), blood (n = 1) and brain abscess (n = 1) between 1983 and 2010; and 48 strains from food such as chicken (n = 8), poultry (n = 3), swine (n = 11), meats (n = 23), vegetables (n = 2) and unknown (n = 1). Samples were collected between 1999 and 2013 from seven States of Brazil including: São Paulo; Santa Catarina; Paraná; Mato Grosso do Sul; Rio Grande do Sul; Goiás; and Bahia (Table 1). Strains were provided by Adolf Lutz Institute of Ribeirao Preto and Oswaldo Cruz Foundation (FIOCRUZ).

**Table 1:** Phenotypic and genotypic resistance profiles of the 90 *Salmonella* Typhimurium strains studied isolated from humans and food in various States between 1983 and 2013 in Brazil.

CFSAN n°	Isolate name	Source	State	Year of isolation	Phenotypic Resistance Profile	Genotypic Resistance Profile (Identity %)						
						Aminoglycoside	Tetracycline	Sulphonamide	Trimethoprim	Beta-lactam	Fluoroquinolone	Phenicol
CFSAN033848	STm01	Human feces	SP	1983	AMP-NA-SXT-STR	*aadA1* (100), *aph(3')-Ia* (99.57)	*tet(C)* (99.92)	―	*dfrA1* (100)	―	―	―
CFSAN033849	STm02	Human feces	SP	1983	AMC-AMP-NA-SXT-C-GEN-STR-SUL	*ant(2")-Ia* (99.06), *aadA1* (100)	―	*sul1* (100)	*dfrA1* (100)	*bla*_OXA-4 (100)_	―	*catA1* (99.85)
CFSAN033850	STm03	Human feces	SP	1983	AMP-NA-SXT-C-GEN-STR-SUL	*aadA1* (100), *aph(3')-Ia* (99.39), *ant(2")-Ia* (99.06)	*tet(C)* (99.92)	*sul1* (100)	*dfrA1* (100)	*bla*_OXA-4 (100)_	―	*catA1* (99.85)
CFSAN033851	STm04	Human feces	SP	1983	AMP-NA-SXT-C-GEN-STR-SUL	*aadA1* (100), *aph(3')-Ia* (99.39), *ant(2")-Ia* (99.06)	―	*sul1* (100)	*dfrA1* (100)	*bla*_OXA-4 (100)_	―	*catA1* (99.85)
CFSAN033852	STm05	Human feces	SP	1983	AMP-NA-SXT-C-GEN-STR-SUL	*aadA1* (100), *aph(3')-Ia* (99.39), *ant(2")-Ia* (99.06)	*tet(C)* (99.92)	*sul1* (100)	*dfrA1* (100)	*bla*_OXA-4 (100),_ *bla*_TEM-187 (98.82)_	―	*catA1* (99.85)
CFSAN033853	STm06	Human feces	SP	1983	―	―	―	―	―	―	―	―
CFSAN033854	STm07	Human feces	SP	1983	AMP-NA-SXT-C-STR	*aph(3')-Ia* (99.25), *aadA1* (100)	―	―	*dfrA1* (100)	*bla*_TEM-1B (100)_	―	*catA1* (99.85)
CFSAN033856	STm09	Human feces	SP	1984	AMP-NA-SXT-C-GEN-SUL	*aadA1* (100), *ant(2")-Ia* (99.06)	―	*sul1* (100)	*dfrA1* (100)	*bla*_OXA-4 (100),_ *bla*_TEM-1B (100)_	―	*catA1* (99.85)
CFSAN033857	STm10	Human feces	SP	1984	NA-SXT-SUL	―	―	―	*dfrA1* (100)	―	―	―
CFSAN033858	STm11	Human feces	SP	1984	AMP-NA-SXT-C-GEN-STR-SUL	*aadA1* (100), *ant(2")-Ia* (99.06)	―	*sul1* (100)	*dfrA1* (100)	*bla*_OXA-4 (100)_	―	*catA1* (99.85)
CFSAN033859	STm12	Human feces	SP	1984	NA-GEN-STR-SUL	*aacA4* (99.64), *aadA1* (100), *aph(3')-Ia* (99.39)	―	*sul1* (100)	*dfrA1* (100)	*bla*_OXA-17 (100)_	*aac(6')Ib-cr* (99.28)	*catA1* (99.85)
CFSAN033860	STm13	Human feces	SP	1984	AMP-NA-SXT-C-GEN-SUL	*aadA1 (100)*, *ant(2")-Ia* (99.06)	―	*sul1* (100)	*dfrA1* (100)	*bla*_OXA-4 (100)_	―	*catA1* (99.85)
CFSAN033861	STm14	Human feces	SP	1984	AMP-NA-SXT	*aph(3')-Ia* (99.39)	*tet(C)* (99.92)	―	*dfrA1* (100)	*bla*_TEM-1B (100)_	―	―
CFSAN033862	STm15	Human feces	SP	1985	SUL	―	―	―	―	―	―	―
CFSAN033863	STm16	Human feces	SP	1985	NA-SXT	*aadA1* (100)	―	―	*dfrA1* (100)	―	―	―
CFSAN033864	STm17	Human feces	SP	1985	―	―	―	―	―	―	―	―
CFSAN033865	STm18	Human feces	SP	1985	―	―	―	―	―	―	―	―
CFSAN033866	STm19	Human feces	SP	1986	AMP-NA-SXT-C-GEN-STR-SUL-CRO	*aacA4* (99.64), *aadA1* (100)	―	*sul1* (100)	*dfrA1* (100)	*bla*_OXA-17 (100)_	*aac(6')Ib-cr* (99.28)	*catA1* (99.85)
CFSAN033867	STm20	Human feces	SP	1986	NA-SXT-C-TET-STR	*aadA1* (100), *aph(3')-Ia* (99.39)	*tet(C)* (99.92)	―	*dfrA1* (100)	―	―	*catA1* (99.85)
CFSAN033868	STm21	Human feces	SP	1986	NA-SXT-STR	*aadA1* (100), *aph(3')-Ia* (99.37)	―	―	*dfrA1* (100)	―	―	―
CFSAN033869	STm22	Human feces	SP	1986	AMC-AMP-NA-SXT-C-GEN-STR-SUL	*aadA1* (100), *aacA4* (99.64)	―	*sul1* (100)	*dfrA1* (100)	*bla*_OXA-17 (100)_	*aac(6')Ib-cr* (99.28)	*catA1* (99.85)
CFSAN033870	STm23	Human feces	SP	1986	TET-STR	*strA* (100), *aph(6)-Id* (100)	*tet(B)* (100)	―	―	―	―	―
CFSAN033871	STm24	Human feces	SP	1986	AMP-NA-SXT-C-GEN-STR-SUL	*aadA1* (100), *aacA4* (99.64)	*tet(C)* (99.92)	*sul1* (100)	*dfrA1* (100)	*bla*_OXA-17 (100)_	*aac(6')Ib-cr* (99.28)	*catA1* (99.85)
CFSAN033872	STm25	Human feces	SP	1986	AMP-NA	*aadA1* (100)	―	―	*dfrA1* (100)	*bla*_TEM-1B (100)_	―	―
CFSAN033873	STm26	Human feces	SP	1986	NA-STR	*aadA1* (100), *aph(3')-Ia* (99.47)	―	―	*dfrA1* (100)	―	―	―
CFSAN033874	STm27	Human feces	SP	1986	AMP-NA-SXT-C-GEN-STR-SUL	*aadA1* (100), *aacA4* (99.64)	*tet(C)* (99.92)	*sul1* (100)	*dfrA1* (100)	*bla*_OXA-17 (100)_	*aac(6')Ib-cr* (99.28)	*catA1* (99.85)
CFSAN033875	STm28	Human feces	SP	1988	SUL	―	―	―	―	―	―	―
CFSAN033876	STm29	Human feces	SP	1989	AMP-STR-SUL	*aph(6)-Id* (100), *aph(3")-Ib* (100)	―	*sul2* (100)	―	*bla*_TEM-1B (100)_	―	―
CFSAN033877	STm30	Human feces	SP	1990	SUL	―	―	―	―	―	―	―
CFSAN033878	STm31	Human feces	SP	1991	SUL	―	―	―	―	―	―	―
CFSAN033879	STm32	Human feces	SP	1992	SUL	―	―	―	―	―	―	―
CFSAN033880	STm33	Human feces	SP	1992	―	―	―	―	―	―	―	―
CFSAN033881	STm34	Human feces	SP	1993	―	―	―	―	―	―	―	―
CFSAN033882	STm35	Human feces	SP	1995	SUL	―	―	―	―	―	―	―
CFSAN033883	STm36	Cold chicken	SP	1995	STR	―	―	―	―	―	―	―
CFSAN033884	STm37	Raw pork sausage	SP	1996	SUL	―	―	―	―	―	―	―
CFSAN033885	STm38	Human feces	SP	1997	SUL	―	―	―	―	―	―	―
CFSAN033886	STm39	Human feces	SP	1998	STR	―	―	―	―	―	―	―
CFSAN033887	STm40	Lettuce	SP	1998	STR-SUL	―	―	―	―	―	―	―
CFSAN033888	STm41	Raw kafta	SP	1998	TET-STR-SUL	*strA* (100), *aph(6)-Id* (100)	*tet(B)* (100)	―	―	―	―	―
CFSAN033889	STm42	Human feces	SP	1999	TET-STR	*strA* (100), *aph(6)-Id* (100)	*tet(B)* (100)	―	―	―	―	―
CFSAN033890	STm43	Human feces	SP	2000	TET-STR	*strA* (100), *aph(6)-Id* (100)	*tet(B)* (100)	―	―	―	―	―
CFSAN033891	STm44	Blood	SP	2000	SUL	―	―	―	―	―	―	―
CFSAN033892	STm45	Raw pork sausage	SP	2000	TET-STR-SUL	*strA* (100), *aph(6)-Id* (100)	*tet(B)* (100)	―	―	―	―	―
CFSAN033893	STm46	Raw tuscan sausage	SP	2002	STR	*strA* (100), *aph(6)-Id* (100)	―	―	―	―	―	―
CFSAN033894	STm47	Human feces	SP	2003	SUL	―	―	―	―	―	―	―
CFSAN033895	STm48	Brain abscess	SP	2005	AMP-SXT-STR-SUL	―	―	*sul2* (100)	*dfrA1* (100)	*bla*_TEM-1B (100)_	―	―
CFSAN033896	STm49	Human feces	SP	2010	NA	―	―	―	―	―	―	―
CFSAN033897	702/99	Final product	SC	1999	―	―	―	―	―	―	―	―
CFSAN033898	12288/06	Swine	SC	2006	AMP-TET-STR-SUL	*strA* (100), *aph(6)-Id* (100)	*tet(B)* (100)	―	―	*bla*_TEM-1B (100)_	―	―
CFSAN033899	12278/06	Swine	SC	2006	NA-TET-STR-SUL	*strA* (100), *aph(6)-Id* (100)	*tet(B)* (100)	―	―	―	―	―
CFSAN033900	12290/06	Swine	SC	2006	TET-STR-SUL	*aph(3")-Ib* (100), *aph(6)-Id* (100)	*tet(B)* (100)	*sul2* (100)	―	―	*oqxA (99*.*40)*, *oqxB* (98.86)	―
CFSAN033901	12268/06	Swine	SC	2006	AMP-NA-STR-SUL	*strA* (100), *aph(6)-Id* (100)	*tet(B)* (100)	―	―	*bla*_TEM-1B (100)_	*oqxA (99*.*40)*, *oqxB* (98.83)	―
CFSAN033902	12381/06	Swine	SC	2006	TET-STR-SUL	*aph(6)-Id* (100), *aph(3")-Ib* (100)	*tet(B)* (100)	*sul2* (100)	―	―	―	―
CFSAN033903	5936/06	Cold chicken	SC	2006	STR-SUL	―	―	―	―	―	―	―
CFSAN033904	5937/06	Cold chicken	SC	2006	SUL	―	―	―	―	―	―	―
CFSAN033905	5934/06	Swine	SC	2006	NA-TET-GEN-STR-SUL	*strA* (100), *aph(4)-Ia* (100),*aph(6)-Id* (100), *aac(3)-IVa* (99.87)	*tet(B)* (100)	―	―	―	*oqxA (99*.*40)*, *oqxB* (98.83)	―
CFSAN033906	5961/06	Swine	SC	2006	TET-GEN-STR-SUL	*aadA1* (99.87)	*tet(B)* (100)	*sul1* (99.89)	―	―	―	―
CFSAN033907	5962/06	Swine	SC	2006	TET-STR-SUL	*aadA1* (99.87)	*tet(B)* (100)	*sul1* (99.89)	―	―	―	―
CFSAN033908	5929/06	Poultry	SC	2006	TET-SUL	―	―	―	―	―	―	―
CFSAN033909	13609/06	Poultry	SC	2006	―	―	―	―	―	―	―	―
CFSAN033910	3848/08	Food	SC	2008	SUL	―	―	―	―	―	―	―
CFSAN033911	16238/09	Ready-to-eat dish	MS	2009	AMP-NA-SXT-C-TET-GEN-STR-SUL	*aac(3)-IIa* (100), *strA* (100), *aph(6)-Id* (100), *aadA1* (99.75)	*tet(A)* (100)	*sul1* (100)	*dfrA1* (100)	*bla*_TEM-1B (100)_	―	*floR* (98.19)
CFSAN033912	16239/09	Ready-to-eat dish	MS	2009	AMP-NA-TET-SUL-CRO	―	*tet(A)* (99.92), *tet(M)* (96.15)	―	―	*bla*_TEM-1B (100)_	―	―
CFSAN033913	16240/09	Ready-to-eat dish	MS	2009	AMP-NA-C-TET-STR-SUL-CRO	―	*tet(A)* (99.92), *tet(M)* (96.15)	―	―	*bla*_TEM-1B (100)_	―	*floR* (98.11)
CFSAN033914	16202/09	Industrialized product	RS	2009	TET-SUL	―	―	―	―	―	―	―
CFSAN033915	16251/09	Industrialized product	GO	2009	AMP-SXT-C-TET-GEN-SUL	*strA* (100), *aph(4)-Ia* (100), *aac(3)-IVa* (99.87), *aph(6)-Id* (100)	*tet(A)* (100), *tet(B)* (100)	*sul1* (100), *sul2* (100)	*dfrA25* (100), *dfrA8* (100)	*bla*_TEM-1A (100)_	*qnrB2* (100)	*floR* (98.19)
CFSAN033916	16273/09	Industrialized product	GO	2009	AMP-NA-TET-GEN-SUL	*aac(3)-IId* (99.88), *aadA2* (100), *aph(3")-Ib* (100), *aph(6)-Id* (100)	*tet(B)* (100)	*sul2* (100)	―	*bla*_TEM-1B (100)_	―	―
CFSAN033917	17307/09	Industrialized product	-	2009	AMP-NA-SXT-TET-GEN-STR-SUL-CRO	*strA* (100), *aac(3)-IIa* (100), *aadA1* (100), *aph(6)-Id* (100)	*tet(A)* (100)	*sul1* (100)	*dfrA1* (100)	*bla*_TEM-1B (100)_	*qnrB88* (100)	―
CFSAN033918	9461/10	In natura meat	SC	2010	SUL	―	―	―	―	―	―	―
CFSAN033919	9479/10	In natura meat	SC	2010	SUL	―	―	―	―	―	―	―
CFSAN033920	7032/10	Poultry	PR	2010	CTX-ATM-FEP-AMP-SXT-TET-STR-SUL-CRO	*strA* (100), *aadA2* (100), *aph(6)-Id* (100)	*tet(B)* (100), *tet(D)* (100)	*sul1* (100), *sul2* (100)	*dfrA12* (100)	*bla*_CTX-M-2 (100)_	―	―
CFSAN033921	3057/10	Frozen chicken carcass	PR	2010	STR-SUL	―	―	―	―	―	―	―
CFSAN033922	6346/10	Chicken	SP	2010	SUL	―	―	―	―	―	―	―
CFSAN033923	5635/10	Unknown	RS	2010	NA	―	―	―	―	―	―	―
CFSAN033924	9109/10	Swine	PR	2010	SUL	―	―	―	―	―	―	―
CFSAN033925	426/10	Chicken	SC	2010	CTX-FEP-AMP-SUL-CRO	―	―	―	―	*bla*_CTX-M-8 (100)_	―	―
CFSAN033926	447/10	Chicken	SC	2010	CTX-FEP-AMP-SUL-CRO	―	―	―	―	*bla*_CTX-M-8 (100)_	―	―
CFSAN033927	2452/11	Frozen chicken carcass	SP	2011	TET-SUL	*aadA2* (100)	*tet(B)* (100)	―	*dfrA12* (100)	―	―	―
CFSAN033928	6709/11	Cold chicken	RS	2011	AMP-NA-SXT-C-TET-GEN-STR-SUL	*aph(6)-Id* (100), *aph(3")-Ib* (100)	*tet(B)* (100)	*sul2* (100)	*dfrA8* (100)	*bla*_TEM-1A (100)_	*oqxA (99*.*40)*, *oqxB* (98.83)	*floR* (98.19)
CFSAN033929	948/12	Raw salad	BA	2012	SUL	―	―	―	―	―	―	―
CFSAN033930	1103/12	Swine (homemade salami)	RS	2012	SUL	―	―	―	―	―	―	―
CFSAN033931	1104/12	Swine (homemade salami)	RS	2012	―	―	―	―	―	―	―	―
CFSAN033932	3330/12	Roast beef	SC	2012	SUL	―	―	―	―	―	―	―
CFSAN033933	994/13	Final product sales (animal origin)	SP	2013	SUL	―	―	―	―	―	―	―
CFSAN033934	374/13	Final product sales (animal origin)	SC	2013	SUL	―	―	―	―	―	―	―
CFSAN033935*	465/13	Final product sales (animal origin)	SP	2013	AMP-SXT-TET-GEN-STR-SUL	*aph(4)-Ia* (100), *aph(3')-Ia* (99.75), *aadA1* (99.87), *aph(3")-Ib* (100), *aac(3)-IVa* (99.87), *aadA15* (96.46), *aph(6)-Id* (100)	*tet(A)* (100), *tet(B)* (100)	*sul1* (100), *sul2* (100)	*dfrA12* (100)	*bla*_TEM-1B (100)_	―	―
CFSAN033937	622/13	Final product sales (animal origin)	SC	2013	NA	―	―	―	―	―	―	―
CFSAN033938	583/13	Final product sales (animal origin)	SC	2013	AMP-TET-SUL	*aadA2* (100)	*tet(A)* (99.92), *tet(M)* (96.15)	―	*dfrA12* (100)	*bla*_TEM-1B (99.88)_	―	―
CFSAN033939	623/13	Final product sales (animal origin)	SP	2013	AMP-NA-C-TET-STR	*aadA1* (100), *aph(3')-Ia* (99.57)	*tet(A)* (100)	―	―	*bla*_TEM-1B (100)_	―	*floR* (98.19)

* This genome was the only one that presented the *mph(A)* (identity 100%) gene that confers resistance to macrolide.

### DNA extraction and quantification

The genomic DNA extraction methods followed Campioni and Falcão [29]. The quality of the DNAs were checked using NanoDrop 1000 (Thermo Scientific, Rockford, IL), and the concentrations were determined using Qubit double-stranded DNA BR assay kit and Qubit fluorometer (Life Technologies, Grand Island, NY) according to each manufacturer’s instructions.

### Genome sequencing, assembly, and annotation

All isolates were prepared using the Nextera Sample Preparation Kit (Illumina, San Diego, CA) and then sequenced on Illumina NextSeq (Illumina) for 2 x 151 cycles. *De novo* assemblies were generated from all raw sequence data. The Illumina reads were assembled with SPAdes 3.0 with the following parameters: only contigs of length ≥500 bp were included; mismatch (MM) 3.28; the genome fraction was 96.157; and number of mis-assemblies (MA) was 2 [30]. The contigs for each isolate (draft genome) were annotated using NCBI’s Prokaryotic Genomes Automatic Annotation Pipeline (PGAAP) [31]. The draft genome sequences of *S*. Typhimurium strains are publicly available in GenBank, with accession numbers listed in S1 Table. The presence of resistance genes, as well as points mutation in the QRDR of the *gyrA*, *gyrB*, *parC*, and *parE* genes, were determined using ResFinder (Center for Genomic Epidemiology, https://cge.cbs.dtu.dk/services/ResFinder/) with settings of threshold of 90%, and minimum length of 60% [32].

### Antimicrobial susceptibility testing

Antimicrobial susceptibility of the 90 *S*. Typhimurium strains were tested by the disc diffusion method of the Clinical and Laboratory Standards Institute (CLSI) [33]. The majority of these results were previously published in Almeida et al. (2015) for 12 antimicrobials including: cefotaxime; cefoxitin; ceftazidime; aztreonam; cefepime; amoxicillin-clavulanic acid; ampicillin; nalidixic acid; levofloxacin; trimethoprim-sulfamethoxazole; chloramphenicol; and ciprofloxacin (Oxoid). However, five additional antimicrobials were tested in this study including: gentamicin; streptomycin; tetracycline; sulfonamides; and ceftriaxone. Additionally, the minimum inhibitory concentrations (MIC) of fluoroquinolones in the nalidixic acid resistant and susceptible strains were evaluated using Etest® following the Clinical and Laboratory Standards Institute (CLSI) guidelines. Strains with MIC ≤ 0.06 μg/mL were considered sensitive and ≥ 1 μg/mL resistant.

### Phylogenetic analysis

In addition to the 90 *S*. Typhimurium strains sequenced in this study, four additional *S*. Typhimurium strains (the sequencing reads were downloaded from NCBI with run accessions of SRR1060710, SRR1963606, SRR6325339, and ERR1556230 for strain DT104, LT2, 14028s, and SL1344, respectively) were added into the phylogenetic analysis for diversity purpose. The genomic analysis was performed using the CFSAN SNP Pipeline that generated the SNP matrix, which was then used to infer the maximum likelihood tree using GARLI [34] with 200 maximum likelihood replicates and 1000 bootstrap iterations. Three samples were included as outgroups including: *Salmonella enterica* serovar Saintpaul CFSAN000611; *Salmonella enterica* serovar Saintpaul CFSAN000614; and *Salmonella enterica* serovar Heidelberg CFSAN000443 [35]. The SNP matrix included 59,130 and 11,176 SNPs, with or without the three outgroups sample, respectively.

## Results

### *In silico* antimicrobial resistance gene analysis

A total of 39 antimicrobial resistance genes were identified (Table 1) and are described in detail below according to the different antimicrobial classes.

#### Aminoglycoside resistance genes

Ten distinct aminoglycoside resistance genes were detected including: the most common gene *aadA1* in 23 (25.6%) isolates (19 humans, 4 foods)*; aph(6)-Id* in 20 (22.2%) isolates (7 humans, 13 foods); *aph(3’)-Ia* in 11 (12.2%) isolates (10 humans, 1 foods); *ant(2”)-Ia* in 7 (7.8%) isolates from humans; *aacA4* in 5 (5.6%) isolates from humans; and *aph(3”)-Ib* in 5 (5.6%) isolates (1 humans, 4 foods); *aph(4)-Ia* in 3 (3.3%) isolates from foods; *aac(3)-IVa* in 3 (3.3%) isolates from foods; and lastly both *aac(3)-IId* and *aadA15* in 1 (1.1%) food isolate each.

#### Tetracycline resistance genes

Five distinct tetracycline resistance genes were detected including: the most common *tet(B)* gene in 19 (21.1%) isolates (3 humans, 16 foods); *tet(A)* in 8 (8.9%) food isolate; *tet(C)* in 7 (7.8%) human isolates; *tet(M)* in 3 (3.3%) food isolates; and *tet(D)* in 1 (1.1%) food isolate.

#### Sulfonamide and trimethoprim resistance

Only two sulfonamide resistance genes were detected including: *sul1* in 19 (21.1%) strains (12 humans 7 foods); and *sul2* in 9 (10%) strains (2 humans 7 foods). The 4 trimethoprim resistance genes detected included: the most common *dfrA1* in 24 (26.7%) isolates (22 human, 2 foods); *dfrA12* in 4 (4.4%) isolates; *dfrA8* in 2 (2.2%) foods; and *dfrA25* in 1 (1.1%) food isolate.

#### Beta-lactam resistance genes

Seven distinct beta-lactam resistance genes were detected including: *bla*_TEM-1B_ in 16 (17.8%) strains (6 human,10 foods); *bla*_OXA-4_ in 7 (7.8%) human isolates; *bla*_OXA-17_ in 5 (5.6%) human isolates; *bla*_TEM-1A_ in 2 (2.2%) food isolates; and *bla*_CTX-M-8_ in 2 (2.2%) food isolates; *bla*_TEM-187_ in 1 (1.1%) human isolate; and *bla*_CTX-M-2_ in 1 (1.1%) food isolate.

#### Fluoroquinolone resistance genes

Five fluoroquinolone resistance genes were detected including: *aac(6')Ib-cr* in 5 (5.6%) human isolates; *oqxA* in 4 (4.4%) food isolates; *oqxB* in 4 (4.4%) food isolates; and *qnrB2* and *qnrB88* each in one (1.1%) food isolate.

#### Phenicol resistance genes

Two phenicol genes were detected including: *catA1* in 14 (15.6%) human isolates; and *floR* in 5 (5.6%) food isolates.

#### Macrolide resistance genes

Only one macrolide resistant gene (*mphA)* was detected in one food isolate.

### Antimicrobial susceptibility testing

A total of 65 (72.2%) out of 90 *S*. Typhimurium strains studied were resistant to sulfonamides, 44 (48.9%) strains were streptomycin resistant, 27 (30%) strains were resistant to tetracycline, 21 (23.3%) strains were gentamicin resistant, and 7 (7.8%) strains were resistant to ceftriaxone. In our previously published paper (26), 34 strains were resistant to nalidixic acid (Nal^R^). In this study we evaluated the reduced susceptibility to fluoroquinolones of 34 strains Nal^R^ and 12 strains susceptible to nalidixic acid (Nal^S^). All the 12 Nal^S^ strains and 21 Nal^R^ strains studied were sensitive to ciprofloxacin (MIC ≤ 0.06 μg/ml), whereas 11 Nal^R^ strains presented intermediate resistance to this drug (MIC 0.12–0.5 μg/ml) and two Nal^R^ strains were resistant to ciprofloxacin. All the antimicrobial susceptibility test results were presented in Table 1.

### Detection of mutations in the *gyrA*, *gyrB*, *parC* and *parE* genes and of the presence of *qnr*, *qepA*, *oqxAB* and *aac(6’)-Ib-cr* genes

A total of 33 (36.7%) out of 90 strains studied presented mutation points in the *gyrA* gene, with all being resistant to nalidixic acid (Table 2). The nonsynonymous points of mutation in the *gyrA* gene included: aspartate/glycine, Asp(87)→Gly in 21 strains; aspartate/asparagine, Asp(87)→Asn in 7 strains; serine/tyrosine, Ser(83)→Tyr in 4 strains; and serine/phenylalanine, Ser(83)→Phe in one strain. None of the strains had more than one mutation point (Table 2). One strain (5934/06 isolated from swine) Nal^R^ did not show mutation in the *gyrA* gene. Seven (7.8%) strains presented synonymous nucleotide mutation, and these strains were Nal^S^ (data not shown) suggesting undiscovered mutations. Thirty-two (35.6%) strains presented synonymous nucleotide mutation in the *parC* gene and 10 of those strains were Nal^R^ with, two strains resistant to ciprofloxacin (data not shown). No strains presented mutations in the *parE* gene.

**Table 2:** Quinolone resistance profiles of the 90 *Salmonella* Typhimurium strains studied isolated from humans and food in various States between 1983 and 2013 in Brazil.

CFSAN n°	Isolate Name	CIP E-test	QRDRs mutations			
			*gyrA* mutation	*gyrB* mutation	*parC* mutation	*parE* mutation
CFSAN033848	STm01	Susceptible	Asp(87)→Gly	―	―	―
CFSAN033849	STm02	Intermediate	Asp(87)→Gly	―	―	―
CFSAN033850	STm03	Susceptible	Asp(87)→Gly	―	―	―
CFSAN033851	STm04	Susceptible	Asp(87)→Gly	―	―	―
CFSAN033852	STm05	Susceptible	Asp(87)→Gly	―	―	―
CFSAN033853	STm06	―	―	―	―	―
CFSAN033854	STm07	Susceptible	Asp(87)→Gly	―	―	―
CFSAN033856	STm09	Susceptible	Asp(87)→Gly	―	―	―
CFSAN033857	STm10	Intermediate	Asp(87)→Gly	―	―	―
CFSAN033858	STm11	Susceptible	Asp(87)→Gly	―	―	―
CFSAN033859	STm12	Susceptible	Asp(87)→Gly	―	―	―
CFSAN033860	STm13	Susceptible	Asp(87)→Gly	―	―	―
CFSAN033861	STm14	Susceptible	Asp(87)→Gly	―	―	―
CFSAN033862	STm15	―	―	―	―	―
CFSAN033863	STm16	Susceptible	Asp(87)→Gly	―	―	―
CFSAN033864	STm17	―	―	―	―	―
CFSAN033865	STm18	―	―	―	―	―
CFSAN033866	STm19	―	Asp(87)→Gly	―	―	―
CFSAN033867	STm20	Susceptible	Asp(87)→Gly	―	―	―
CFSAN033868	STm21	Susceptible	Asp(87)→Gly	―	―	―
CFSAN033869	STm22	Susceptible	Asp(87)→Gly	―	―	―
CFSAN033870	STm23	―	―	―	―	―
CFSAN033871	STm24	Susceptible	Asp(87)→Gly	―	―	―
CFSAN033872	STm25	Susceptible	Asp(87)→Gly	―	―	―
CFSAN033873	STm26	Susceptible	Asp(87)→Gly	―	―	―
CFSAN033874	STm27	Susceptible	Asp(87)→Gly	―	―	―
CFSAN033875	STm28	Susceptible	―	―	―	―
CFSAN033876	STm29	Susceptible	―	―	―	―
CFSAN033877	STm30	―	―	―	―	―
CFSAN033878	STm31	Susceptible	―	―	―	―
CFSAN033879	STm32	―	―	―	―	―
CFSAN033880	STm33	―	―	―	―	―
CFSAN033881	STm34	Susceptible	―	―	―	―
CFSAN033882	STm35	Susceptible	―	―	―	―
CFSAN033883	STm36	Susceptible	―	―	―	―
CFSAN033884	STm37	Susceptible	―	―	―	―
CFSAN033885	STm38	―	―	―	―	―
CFSAN033886	STm39	―	―	―	―	―
CFSAN033887	STm40	Susceptible	―	―	―	―
CFSAN033888	STm41	―	―	―	―	―
CFSAN033889	STm42	―	―	―	―	―
CFSAN033890	STm43	―	―	―	―	―
CFSAN033891	STm44	Susceptible	―	―	―	―
CFSAN033892	STm45	Susceptible	―	―	―	―
CFSAN033893	STm46	Susceptible	―	―	―	―
CFSAN033894	STm47	Susceptible	―	―	―	―
CFSAN033895	STm48	―	―	―	―	―
CFSAN033896	STm49	Intermediate	Asp(87)→Asn	―	―	―
CFSAN033897	702/99	―	―	―	―	―
CFSAN033898	12288/06	―	―	―	―	―
CFSAN033899	12278/06	Susceptible	Asp(87)→Asn	―	―	―
CFSAN033900	12290/06	―	―	―	―	―
CFSAN033901	12268/06	Intermediate	Asp(87)→Asn	―	―	―
CFSAN033902	12381/06	―	―	―	―	―
CFSAN033903	5936/06	―	―	―	―	―
CFSAN033904	5937/06	―	―	―	―	―
CFSAN033905	5934/06	Susceptible	―	―	―	―
CFSAN033906	5961/06	―	―	―	―	―
CFSAN033907	5962/06	―	―	―	―	―
CFSAN033908	5929/06	―	―	―	―	―
CFSAN033909	13609/06	―	―	―	―	―
CFSAN033910	3848/08	―	―	―	―	―
CFSAN033911	16238/09	Resistant	Ser(83)→Tyr	―	―	―
CFSAN033912	16239/09	Intermediate	Asp(87)→Asn	―	―	―
CFSAN033913	16240/09	Intermediate	Asp(87)→Asn	―	―	―
CFSAN033914	16202/09	―	―	―	―	―
CFSAN033915	16251/09	―	―	―	―	―
CFSAN033916	16273/09	Intermediate	Ser(83)→Phe	―	―	―
CFSAN033917	17307/09	Resistant	Ser(83)→Tyr	―	―	―
CFSAN033918	9461/10	―	―	―	―	―
CFSAN033919	9479/10	―	―	―	―	―
CFSAN033920	7032/10	―	―	―	―	―
CFSAN033921	3057/10	―	―	―	―	―
CFSAN033922	6346/10	―	―	―	―	―
CFSAN033923	5635/10	Intermediate	Asp(87)→Asn	―	―	―
CFSAN033924	9109/10	―	―	―	―	―
CFSAN033925	426/10	―	―	―	―	―
CFSAN033926	447/10	―	―	―	―	―
CFSAN033927	2452/11	―	―	―	―	―
CFSAN033928	6709/11	Intermediate	Asp(87)→Asn	―	―	―
CFSAN033929	948/12	―	―	―	―	―
CFSAN033930	1103/12	―	―	―	―	―
CFSAN033931	1104/12	―	―	―	―	―
CFSAN033932	3330/12	―	―	―	―	―
CFSAN033933	994/13	―	―	―	―	―
CFSAN033934	374/13	―	―	―	―	―
CFSAN033935	465/13	―	―	―	―	―
CFSAN033937	622/13	Intermediate	Ser(83)→Tyr	―	―	―
CFSAN033938	583/13	―	―	―	―	―
CFSAN033939	623/13	Intermediate	Ser(83)→Tyr	―	―	―

The *qnrB88* gene was found in 1 (1.1%) Brazilian strain that previously had been reported both in *Klebsiella pneumoniae* (GenBank: KX118608) and under another gene (*qnrE1)* found in *Klebsiella pneumonia* (GenBank: KY781949). Additionally, one strain had the *qnrB2* gene present in *Salmonella* Bredeney (GenBank: FJ844401). The *oqxAB* gene was found in 4 (4.4%) strains. However, these genes diverged in having 6 mutations compared to the *oqxAB* of *Salmonella* Derby (GenBank: FN811184). The *aac(6’)Ib-cr* gene was identified in 5 strains isolated from humans.

### Phylogenetic analysis

**Fig 1:**
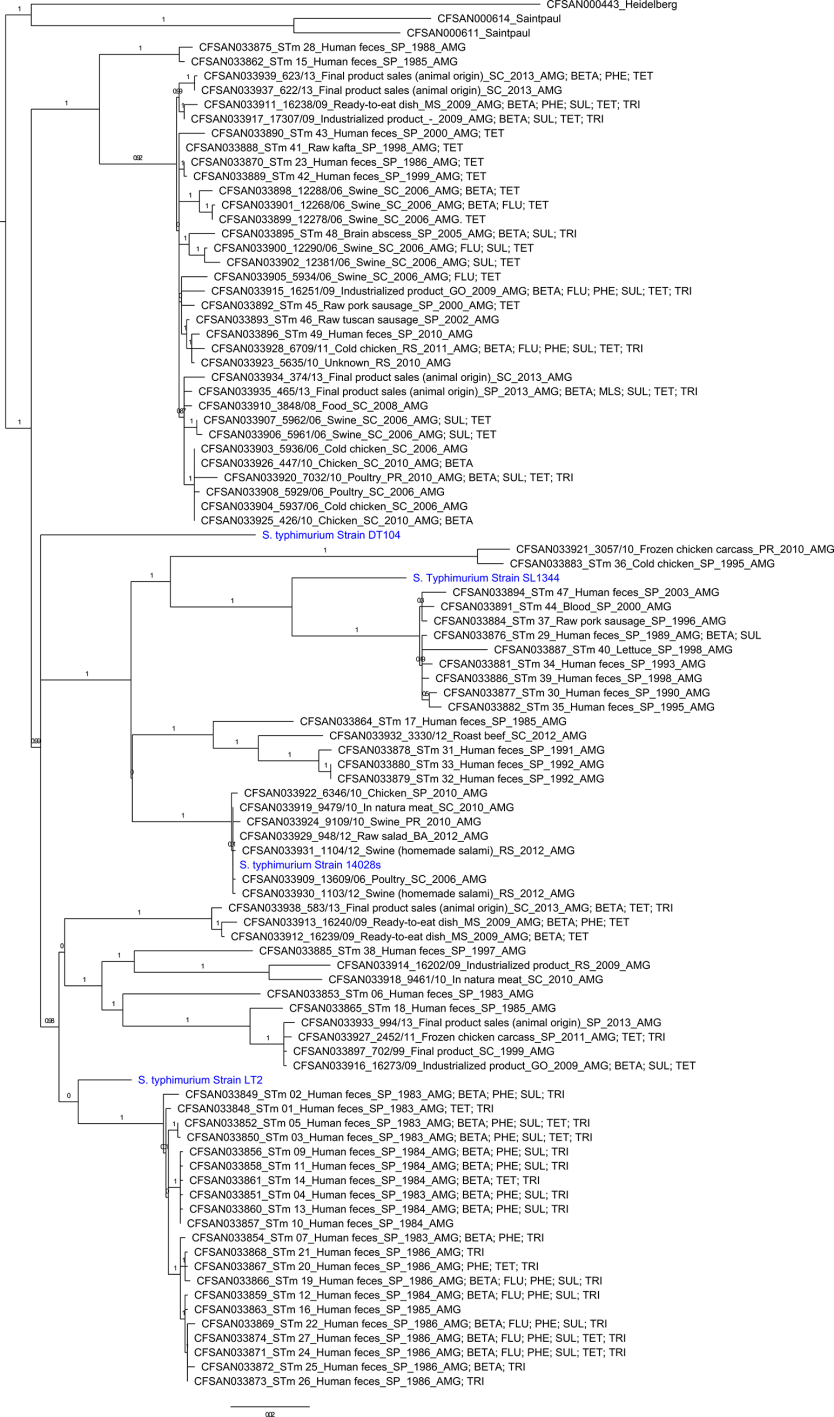
Phylogenetic analysis based on SNPs of the 90 *Salmonella* Typhimurium strains of this study and four additional *S*. Typhimurium strains (the sequencing reads were downloaded from NCBI with run accessions of SRR1060710, SRR1963606, SRR6325339, and ERR1556230 for strain DT104, LT2, 14028s, and SL1344, respectively). The genomes of one *Salmonella* Heidelberg and two *Salmonella* Saintpaul were used as outgroup.

The 90 *S*. Typhimurium strains studied were distributed into 2 major clades (designated A and B, Fig 1). Clade A comprised 34 (37.8%) strains with 7 isolated from humans between 1985 and 2010, and 27 isolated from food between 1998 and 2013. Thirty-four strains located in Clade A were isolated from South, Southeast and Midwestern Regions in Brazil. Of the 34 strains in Clade A, 15 strains (14 foods, 1 human) were resistant to three or more antimicrobial classes being multidrug-resistant (MDR). Clade B comprised 56 (62.2%) strains with 35 isolated from humans between 1983 and 2003, and 21 strains isolated from food between 1995 and 2013. Fifty-six strains located in Clade B were from South, Southeast, Northeast and Midwestern Regions in Brazil. Of the 56 strains in Clade B, 23 strains (18 humans, 5 foods) were MDR. All reference genomes added were grouped in clade B (DT104, SL1344, 14028s and LT2).

## Discussion

In this study 90 *S*. Typhimurium strains isolated from food and humans in Brazil were sequenced by next generation sequencing technology to evaluate their antimicrobial resistance gene profiles and phylogenetic diversity. This is the first study of *S*. Typhimurium strains isolated in Brazil that used WGS to access the genetic diversity and the molecular bases of antimicrobial resistance. In previous studies, the same strains were typed by PFGE, MLVA, ERIC-PCR, CRISPR-MVLST and MLST [26–28].

In this study, 47 (52.2%) strains presented phenotypic resistance to gentamicin and/or streptomycin. Streptomycin is not frequently used to treat *Salmonella enterica* infections; but, it has been commonly used as a growth promoter in food-producing animals and for this reason may serve as a marker for resistant strains moving through the food supply [11].

Our results confirm McDermott et al’s. [11] observations of discrepancies between phenotypic resistance and genotypic resistance of aminoglycoside resistant genes. We observed 35 isolates carrying streptomycin resistance genes, but these isolates were phenotypically susceptible to the drugs. It is unclear why the genes while present in the genomes were not expressed to provide phenotypic resistance. Presence of the known streptomycin resistance genes does not predict phenotypic resistance well for this class.

The tetracycline resistance genes were found in 32 (35.5%) strains. Interestingly, 2 strains that were phenotypically resistant to tetracycline did not present any known tetracycline resistance genes suggesting a possible alternative mode of resistance. In contrast, seven strains that presented tetracycline resistance genes were phenotypically susceptible. Of these seven, six strains had two tetracycline resistance genes and one strain had only one tetracycline resistance gene. Tetracycline has been used commonly as an antibiotic in swine husbandry [36]. Brazil is a major producer of pigs with 3.73 million tons of pork produced and exported in 2016 [37, 38]. The *Salmonella* Typhimurium serovar usually does not cause severe disease in pigs and sometimes it is asymptomatic in these animals, which may be a serious public health problem, since it may be an important source of contamination of carcasses in slaughterhouses. In addition, the contamination by *S*. Typhimurium may not be detected while the pigs are on the farm, which may eventually lead to human contamination [36, 39].

Cefoxitin resistance has been used to indicate certain types of beta-lactamases production by *Salmonella* and *E*. *coli*. First and second-generation cephalosporin susceptibility results are not reported in clinical medicine for *Salmonella*, because the drugs may appear active *in vitro*, but are not therapeutically effective [33]. Regarding the beta-lactam resistance genes found in Brazil, the most common was *bla*_TEM-1B_ gene presented in 16 (17.8%) isolates (6 humans, 10 foods). The *bla*_TEM-1B_ gene has been associated with ampicillin resistance and 32 (35.6%) strains were phenotypically resistant to the ampicillin. The *bla*_CTX-M-8_ and *bla*_CTX-M-2_ genes have been more closely associated to cephalosporin resistance and 7 strains were resistant to ceftriaxone (CRO), third generation cephalosporin, but only 3 strains presented a *bla*_CTX_ allele. The most common resistant gene was *aac(6')Ib-cr* found in 5 (5.6%) human isolates followed by *oqxA* and *oqxB* found in 4 (4.4%) food isolates. The *qnrB2* and *qnrB88* genes were found each in 1 (1.1%) food isolate.

Some of the discrepancies observed when a resistance gene is present but no phenotypic resistance in bacterial growth is observed, or when the phenotype is present but no known resistance gene is observed, is likely due to new unidentified resistance genes or mutations conferring resistance in undiscovered genes. Therefore, it is important to study any discrepancy as each represents new ways that bacteria are acquiring resistance as was reported for a new mechanisms discovered for *Campylobacter* gentamicin resistance [40]. Pribul et al. [41] evaluated the prevalence of PMRQ genes in 129 isolates of non-typhoidal *Salmonella* from Brazil by PCR amplification. *Qnr* genes were found in 15 (11.6%) isolates (8 *qnrS*, 6 *qnrB*, and 1 *qnrD*), and the *aac(6′)-Ib* gene was found in 23 (17.8%) isolates. Regarding mutation points in the QRDRs, *gyrA* mutation was the only one found among the strains studied. Thirty-three (36.7%) of nalidixic acid resistant strains presented mutations in the *gyrA* gene (22 human, 11 foods).

McDermott and colleagues [11] used WGS technology to identify known antimicrobial resistance genes among 640 non-typhoidal *Salmonella* strains for 43 different serotypes and correlated these with susceptibility phenotypes to evaluate the utility of WGS for antimicrobial resistance surveillance. Overall, genotypic and phenotypic resistance correlated in 99.0% of the cases. They concluded that WGS is an effective tool for predicting antibiotic resistance in non-typhoidal *Salmonella* [11]. Regarding QRDR mutations and PMQR genes, 21 isolates had either QRDR mutations or PMQR genes, all of which were from human clinical cases. In contrast, in this study QRDR mutations were found in both human and food isolates.

*Salmonella* Typhimurium ST313 had been described only in sub-Saharan Africa, with high levels of antibiotic resistance associated with bloodstream infections and mortality rates of >25% [42, 43]. In 2017 [28], nine strains were typed as ST313 in Brazil, with only 1 MDR, human strain (STm29 feces), presenting resistant to ampicillin, streptomycin and sulfonamide. Five Brazilian strains (STm30, STm35, STm37, STm47, STm44) were resistant just to sulfonamide with STm37 isolated from food. Other resistant strains included: STm40 isolated from food (streptomycin and sulfonamide); STm39 isolated from human feces (streptomycin); and STm34 isolated from human feces (pan_susceptible).

Food isolates were distributed in Clades A and B in relatively similar numbers suggesting that there is more than one subtype in circulation, in foods in Brazil. Human’s isolates were more prevalent in the Clade B suggesting the existence of a prevalent subtype. Genomic and phenotypic testing results suggest clinical strains isolated before the mid-1990s presented more antimicrobial resistance compared to later strains. The diversity and prevalence of resistant genes found in Brazilian *Salmonella* Typhimurium is an alert of their potential hazard for food safety and public health.

## Supporting information

S1 Table Characteristics of the 90 *Salmonella* Typhimurium genomes studied. (XLSX)
